# The Associations of Platelet Activation and Coagulation Parameters with Obstructive Sleep Apnoea: A Large-Scale Observational Study

**DOI:** 10.1155/2023/5817644

**Published:** 2023-02-27

**Authors:** Jundong Yang, Wenjun Xue, Zhicheng Wei, Caiqiong Hou, Huaming Zhu, Huajun Xu, Xiaolin Wu, Yunhai Feng, Xinyi Li

**Affiliations:** ^1^Central Laboratory of Shanghai Eighth People's Hospital Affiliated to Jiangsu University, Shanghai, China; ^2^Department of Otorhinolaryngology Head and Neck Surgery, Shanghai Eighth People's Hospital Affiliated to Jiangsu University, Shanghai, China; ^3^Department of Otolaryngology Head and Neck Surgery & Center of Sleep Medicine, Shanghai Jiao Tong University Affiliated Sixth People's Hospital, Yishan Road 600, 200233 Shanghai, China; ^4^Shanghai Key Laboratory of Sleep Disordered Breathing, Shanghai, China; ^5^Basic Medical College, Jiamusi University, Jiamusi 154007, Heilongjiang, China

## Abstract

**Objectives:**

Obstructive sleep apnoea (OSA) is associated with an increased risk of cardiovascular disease, with alterations in coagulability suspected as the mediating factor. This study explored blood coagulability and breathing-related parameters during sleep in patients with OSA.

**Design:**

Cross-sectional observational study. *Setting*. Shanghai Sixth People's Hospital. *Participants.* 903 patients diagnosed by standard polysomnography. *Main Outcome and Measures.* The relationships between coagulation markers and OSA were evaluated using Pearson's correlation, binary logistic regression, and restricted cubic spline (RCS) analyses.

**Results:**

The platelet distribution width (PDW) and activated partial thromboplastin time (APTT) decreased significantly with increasing OSA severity (both *p* < 0.001). PDW was positively associated with the apnoea-hypopnea index (AHI), oxygen desaturation index (ODI), and microarousal index (MAI) (*ß* = 0.136, *p* < 0.001; *ß* = 0.155, *p* < 0.001; and *ß* = 0.091, *p* = 0.008, respectively). APTT was negatively correlated with AHI (*ß* = −0.128, *p* < 0.001) and ODI (*ß* = −0.123, *p* = 0.001). PDW was negatively correlated with percentage of sleep time with oxygen saturation below 90%(CT90) (*ß* = −0.092, *p* = 0.009). The minimum arterial oxygen saturation (SaO_2_) correlated with PDW (*ß* = −0.098, *p* = 0.004), APTT (*ß* = 0.088, *p* = 0.013), and prothrombin time (PT) (*ß* = 0.106, *p* = 0.0003). ODI was risk factors for PDW abnormalities (odds ratio (OR) = 1.009, *p* = 0.009) after model adjustment. In the RCS, a nonlinear dose-effect relationship was demonstrated between OSA and the risk of PDW and APTT abnormalities.

**Conclusion:**

Our study revealed nonlinear relationships between PDW and APTT, and AHI and ODI, in OSA, with AHI and ODI increasing the risk of an abnormal PDW and thus also the cardiovascular risk. This trial is registered with ChiCTR1900025714.

## 1. Introduction

Obstructive sleep apnoea (OSA) is a chronic disease characterised by recurrent partial or complete upper airway collapse, resulting in a temporary pause of breathing during sleep and thus intermittent oxygen desaturation [1]. It is an independent risk factor for a hypercoagulable state and arterial thrombosis, both of which contribute to the development of cardiovascular disease (CVD) [2, 3].

Platelet activation and coagulation, blood viscosity, haematocrit, plasma fibrinogen, and other potential thrombosis markers are increased in patients with OSA [4]. A marker of thrombosis, and thus of platelet activation, is the platelet distribution width (PDW), which reflects the variance in the size of circulating platelets and is better standardized than the mean platelet volume (MPV) [5]. The prothrombin time (PT) and activated partial thromboplastin time (APTT) are used to evaluate intrinsic and extrinsic pathways of coagulation [6]. Changes in either pathway indicate fluctuations in blood coagulability [7]. An increased PDW and decreased APTT are characteristic of OSA [8], and the haematological parameters of patients with OSA improved significantly after treatment [9]. However, the small sample size and the use of nonstandard PSG in most studies have produced incomplete and inaccurate findings.

We explored the relationship between coagulation parameters and OSA in a large cohort. Our study was limited to male participants, as previous studies investigating sex-based differences in OSA [1], venous thromboembolism [10], and CVD [11] identified a higher prevalence of OSA in males than females, as well as an increased risk of coagulation disorders, and thus a higher risk of CVD and thromboembolism.

## 2. Methods

### 2.1. Subjects

Participants with suspected OSA seen between 2013 and 2018 were enrolled in the study. The inclusion criteria were whole-night PSG and a haematological examination; age ≥18 years; and male gender. The 1,352 initially enrolled patients were then screened according to the following exclusion criteria: previously diagnosed with OSA and treated with continuous positive airway pressure (CPAP), oral orthotics, upper airway surgery, etc.; systemic diseases, such as chronic liver disease, renal insufficiency, hyperthyroidism, hypothyroidism, or tumour; mental or neurological disorders; alcoholism; blood or platelet donation in the last 6 months; regular use of drugs affecting coagulation, such as aspirin, clopidogrel hydrogen sulphate tablets, and low-molecular heparin; and other sleep disorders, such as restless legs syndrome and narcolepsy. Ultimately, 903 male patients were included in this cross-sectional observational study.

The study was conducted in accordance with the Declaration of Helsinki. The Ethics Committee approved this study, and the trial was registered (ChiCTR1900025714) prior to commencement. Informed consent was obtained from all participants.

### 2.2. Polysomnographic Evaluation

Parameters related to breathing during sleep were assessed by overnight PSG (Alice 4 or 5; Respironics, Pittsburgh, PA, USA) and manually scored according to the guideline of the American Academy of Sleep Medicine 2012 [12]. Apnoea was defined as complete cessation or at least 90% deduction of airflow lasting for ≥10 s, and hypopnea as a ≥ 30% reduction accompanied by either a ≥ 3% decrease in oxyhaemoglobin saturation or an associated arousal for ≥10 s. The apnoea-hypopnea index (AHI) was defined as the number of apnoea and hypopnea events per hour during the total sleep time. The oxygen desaturation index (ODI) was calculated as the total number of episodes of ≥4% oxyhaemoglobin desaturation during sleep. The microarousal index (MAI) was defined as the mean number of arousals per hour of sleep. The arterial oxygen saturation (SaO_2_, %) was monitored by pulse oximetry during sleep, then the percentage of sleep time with oxygen saturation below 90% (CT90) was calculated according to the data of monitor time, and SaO_2_. OSA severity was classified based on the AHI, as follows: non-OSA, AHI < 5; mild OSA, 5 ≤ AHI < 15; moderate OSA, 15 ≤ AHI < 30; and severe OSA, AHI ≥ 30.

### 2.3. Anthropometric Measurements and Coagulation Tests

Height and weight were measured using a digital scale, with patients in a standing position and dressed in light clothing with bare feet. Neck circumference (NC) was measured at the middle of the cricothyroid membrane, waist circumference (WC) midway between the lowest rib and iliac crest, and hip circumference (HC) at the largest gluteal circumference. The mean values of the measurements were used in the analysis. BMI was calculated as weight in kilograms divided by height in meters squared (kg/m^2^). The weight-to-height ratio (WHR) was calculated as WC/HC. Smoking or drinking was defined as subjects who were self-reported to smoke or drink.

Venous blood samples were collected from patients in the morning following the overnight PSG. Ethylenediaminetetraacetic acid (EDTA) and sodium citrate were used as anticoagulants for platelet and coagulation tests, respectively. The platelet count (PLT), MPV and PDW, were measured using an XN-3000 analyser (SYSMEX, Hyogo, Japan) at optimal measurement time. The coagulation tests included the APTT, thrombin time (TT), and PT, all measured using a CS-5100 analyser (SYSMEX). An abnormal PDW was defined as <9.8% or >16.2%, an abnormal APTT as <20 or >40 seconds, and an abnormal TT as <13 or >21 seconds according to the diagnostic criteria of the Guidelines on the Laboratory Aspects of Assays used in hemostasis and thrombosis [13].

### 2.4. Statistical Analysis

All statistical analyses were performed using SPSS (version. 21.0; IBM Corp., Armonk, NY, USA) and MATLAB 8.0 (MathWorks Corp., Natick, MA, USA) software. Data on the general characteristics of the participants are presented as the median and interquartile range (25–75%). *P*-values for linear trends across different groups were calculated using the polynomial linear trend test for continuous variables. Bivariate correlation analyses were used to explore the relationships between sleep-breath parameters (AHI, ODI, MAI, CT90, and the minimum SaO_2_) and the coagulation indicators (PLT, PDW, MPV, APTT, PT, and TT), while binary logistic regression analyses were performed to evaluate the relationships of AHI, ODI, MAI, CT90, and minimum SaO_2_ with the risk of coagulation. Covariates including age, BMI, WHR, smoking, drinking, and hypertension were adjusted in different models: model 1 (age, BMI); model 2 (variables included in model 1 and hypertension); model 3 (age, WHR, and hypertension); model 4 (smoking and drinking based on model 2); and model 5 (variables in model 4 and additional WHR). Nonlinear relationships between OSA and coagulation indicators were evaluated in a restricted cubic spline (RCS) analysis. Knots for the AHI, ODI, and minimum SaO_2_ were identified in the RCS analysis using R software (R Development Core Team, Vienna, Austria). Two-sided*p*-values <0.05 were considered to indicate statistical significance.

## 3. Results

### 3.1. Baseline Characteristics

The final study group consisted of 101 non-OSA, 120 mild OSA, 147 moderate OSA, and 535 severe patients with OSA. The basic anthropometric and haematological characteristics are reported in Table 1. BMI, NC, WC, HC, WHR, smoking, and drinking differed significantly according to OSA severity, tending to show an increase with more severe OSA (*p* for linear trend <0.001). PDW increased, and APTT decreased significantly, with increasing OSA severity (both *p* < 0.001). The associations of MPV, TT, and PT and OSA severity were not significant (*p* > 0.05).

### 3.2. Associations between Breathing during Sleep and Coagulation Parameters

Pearson correlation analysis showed that AHI, ODI, and MAI were significantly positively associated with PDW (*ß* = 0.136, *p* < 0.001; *ß* = 0.155, *p* < 0.001; and ß = 0.091, *p*=0.008, respectively; Table 2). The minimum SaO_2_ and CT90 were negatively associated with PDW (*ß* = −0.098, *p*=0.004; and ß = −0.092, *p*=0.009, respectively). APTT (*ß* = 0.088, *p*=0.013) and PT (*ß* = 0.106, *p*=0.003) were positively associated with minimum SaO_2_. APTT was negatively associated with AHI and ODI (*ß* = −0.128, *p* < 0.001; and ß = −0.123, *p*=0.001, respectively). There were no associations between TT and OSA-related parameters (Table 2).

AHI, ODI, and minimum SaO_2_ were associated significantly with the risk of coagulation while ODI, MAI, and CT90 were not (Table 3). AHI and ODI increased the risk of an abnormal PDW after adjustments in model 1 (OR = 1.008, 95% CI: 1.002–1.015, *p*=0.015; OR = 1.010, 95% CI: 1.003–1.016, *p*=0.003, respectively), and the significance still existed after further adjusting for hypertension, smoking, and drinking (model 4 (OR = 1.008, 95% CI: 1.001–1.015, *p*=0.022; OR = 1.009, 95% CI: 1.003–1.016, *p*=0.005, respectively)). In model 5 adjusting for all potential confounders, ODI increased the risk of an abnormal PDW (odds ratio (OR) = 1.009, 95% confidence interval (95% CI): 1.002–1.016, *p*=0.009), and minimum SaO_2_ increased the risk of an abnormal TT (OR = 1.050, 95% CI: 1.007–1.094, *p*=0.021).

### 3.3. Nonlinear RCS Analysis

**Figure 1:**
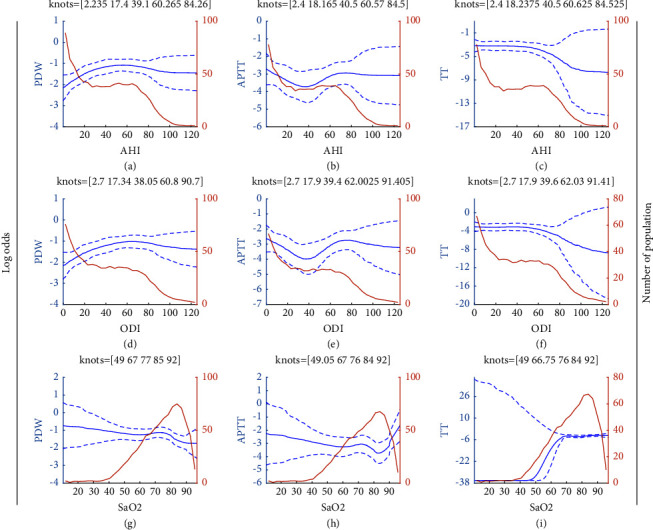
Restricted cubic spline regression of the nonlinear relationship between abnormal coagulation (including platelet distribution width (PDW), activated partial thromboplastin time (APTT), and thrombin time (TT) data) and the severity of obstructive sleep apnoea (OSA). The *x*-axis represents the continuous values of the apnoea-hypopnea index (AHI), oxygen desaturation index (ODI), or arterial oxygen saturation (SaO_2_). The left *y*-axis represents the log odds of abnormal coagulation for each coagulation marker. The right *y*-axis represents the number of patients.

To assess the dose-effect relationship between OSA and the risk of hypercoagulability/hypocoagulability (abnormal coagulation), AHI, ODI, and the minimum SaO_2_ were analysed as continuous variables (Figure 1, *x*-axis) and the log odds of PDW, APTT, and TT as categorical variables (Figure 1, *y*-axis). The risk of abnormal coagulation did not always increase with increasing OSA severity. The relationships of AHI with abnormal PDW (Figure 1(a)) and APTT (Figure 1(b)) were not linear. In the RCS, the knots for AHI and log odds of an abnormal PDW for patients with OSA with an AHI ≤84.26 were 2.235, 17.4, 39.1, and 84.26, respectively, and reached a plateau. Knots for AHI of 2.4, 18.165, and 40.5 indicated a decreased risk of an abnormal APTT, as reflected in the slopes of the curves (Figure 1(a)). However, the risk of an abnormal APTT increased in patients with an AHI >60.57, and a plateau was reached at an AHI of 84.5 (Figure 1(b)). The same trend was observed for AHI and ODI; for patients at the risk of an abnormal PDW, the ODI increased, followed by a plateau (Figure 1(d)). For patients at the risk of an abnormal APTT, the ODI declined, followed by a rise and then a plateau (Figure 1(e)). The relationship between the risk of an abnormal TT and OSA was close to linear (Figures 1(c), 1(f), and 1(i)).

## 4. Discussion

This study explored the associations between breathing-related variables and haematological parameters related to coagulation. The results showed that PDW and APTT were associated with OSA-related traits, especially the AHI and ODI, which are risk factors for an abnormal PDW. Both nonlinear and nonmonotonic relationships between OSA and coagulation were determined.

The size distribution of platelets in the peripheral circulation is expressed as the PDW, which is considered a marker of thromboembolic diseases [14]. Fan et al. [15] reported a significantly higher PDW in patients with severe OSA. A meta-analysis also demonstrated that OSA was associated with a high PDW [8]. The significant correlations of the PDW and AHI with the minimum SaO_2_ in that study were consistent with our results [16]. Children with OSA also have a higher platelet count and higher PDW [17]. In another study, the PDW improved significantly in patients with OSA after CPAP treatment [9]. Our results also indicated a correlation of the PDW with ODI, CT90, and MAI, with ODI independently increasing the risk of an abnormal PDW. This observation suggested that PDW is affected by hypoxia and thus serves as a meaningful blood marker in patients with OSA. Elevated surface marker expression in significantly hypoxemic OSA individuals is consistent with the platelet activation seen in this group.

In addition to platelet activation, thrombosis is modulated by the coagulation system and thus related to CVD [18]. PT, TT, and APTT are indicators of extrinsic, common, and intrinsic coagulation pathways, respectively [6, 7]. A previous study showed that APTT correlated with ODI in children with OSA, which suggested that OSA-related traits enhance coagulability in paediatric OSA [19]. PT was also shown to decrease with increased OSA severity and correlated with AHI [20]. Although in our study, there was no significant difference in PT as a function of OSA severity, we did find that PT was positively associated with the minimum SaO_2_. Previous findings of an improvement in PT, TT, and APTT in patients with OSA after CPAP treatment [9] and upper airway surgery thus indicate that improved ventilation and the correction of hypoxia improve coagulation parameters in OSA.

In our study, several potential confounders that might affect coagulation were adjusted, and a significant association of hypoxia-related parameters with the risk of coagulation still existed, revealing the associations between OSA and platelet activation and coagulation system. Chronic intermittent hypoxia is the hallmark of OSA, but the underlying mechanism of platelet activation and disturbed coagulation in response to hypoxic conditions remains to be determined. Krieger et al. [21] reported that intermittent hypoxemia in OSA suppressed platelet responses to epinephrine and thrombin and decreased the level of the surface marker CD40L, thus signifying increased platelet activation, and it was also demonstrated that platelet activation in hypoxic mice can contribute to hypoxia-induced inflammation [22]. CAPNS1-dependent calpain, activated during the platelet activation cascade, is associated with hypoxia-induced thrombogenesis [23]. Hypoxia acts on the platelet purinergic signaling pathway by increasing P2Y1-receptor and ADP pathway activities, which is of potential therapeutic interest [24]. OSA was shown to elevate circulating levels of platelet-derived microparticles released by platelet activation, thus increasing the risk of CVD [25]. Moreover, such platelet-derived microparticles with CD41^+^ and annexin V^+^ variated diurnally, increasing from morning and reaching their peak levels in the afternoon [26], suggesting a more complicated link between platelet activation and the risk of CVD in patients with OSA. Few studies have focused on the intrinsic coagulation pathway in OSA and hypoxia. Consequently, the molecular mechanisms underlying platelet activation and coagulation in OSA and its complications are not fully understood, and circadian rhythm could be considered as a potential aspect in future studies.

Our study also revealed nonlinear relationships of OSA-related traits with PDW and APTT, highlighting the complex role of the coagulation system in OSA. The reciprocal interactions of platelets and the coagulation system, and of plasma coagulants and blood cells, are crucial aspects of thrombus formation and are also keys to elucidating the pathophysiology of thrombosis in many diseases, including OSA [27]. Since adherence to CPAP therapy is limited, further studies of the clinical utility of coagulation indices for monitoring patients with OSA are needed.

Our study had several limitations. Firstly, due to its cross-sectional design, causal relationships could not be determined. Secondly, some haematology markers, such as blood viscosity and clotting factors, were not evaluated. Thirdly, the roles played by environmental factors, such as diet, exercise frequency, and economic conditions, were not considered. Further research is therefore needed to understand the effects of intermittent hypoxia on blood viscosity, fibrinolysis system, and other factors contributing to the hypercoagulation state of patients.

## 5. Conclusion

This study demonstrated associations of PDW and APTT with OSA-related traits and showed that AHI and ODI are risk factors for an abnormal PDW. The nonlinear relationship between platelet activation and coagulation parameters with OSA provided evidence of the complex interactions among platelets, coagulation, and hypoxia, which may contribute to the cardiovascular complications seen in patients with OSA.

## Figures and Tables

**Table 1:** Basic characteristics of the overall population by the severity of OSA.

Characteristics	Non-OSA (*N* = 101)	Mild OSA (*N* = 120)	Moderate OSA (*N* = 147)	Severe OSA (*N* = 535)	*P* for linear trend
Age (years)	47 (47–60)	60 (47–62)	47 (47–62)	47 (47–60)	0.239
BMI (kg/m^2^)	25.7 (22.7–29.3)	26.7 (23.4–30.0)	27.6 (25.0–30.2)	28.7 (26.0–30.9)	**<0.001**
NC (cm)	39 (35–41)	40 (37–42)	41 (38–43)	41 (40–44)	**<0.001**
WC (cm)	93 (84–102)	96 (87–106)	99 (92–107)	101 (95–108)	**<0.001**
HC (cm)	99 (91–104)	101 (95–107)	102 (97–108)	103 (99–109)	**<0.001**
WHR	0.94 (0.89–0.99)	0.96 (0.91–0.99)	0.96 (0.93–1.01)	0.97 (0.94–1.01)	**<0.001**
Smoking (*n* (%))	34 (34.7)	34 (30.1)	69 (47.9)	303 (59.4)	**<0.001**
Drinking (*n* (%))	36 (36.7)	39 (34.5)	77 (53.5)	302 (59.3)	**<0.001**

*Haematological parameters*					
PLT (×10^3^/*μ*L)	223 (180–273)	219 (186–260)	206 (178–256)	220 (183–257)	**0.046**
MPV (fL)	10.7 (10–11.5)	10.6 (9.9–11.4)	10.6 (10–11.5)	10.6 (9.9–11.5)	0.97
PDW (fL)	13 (11.3–14.6)	12.7 (11.4–14.2)	13.1 (11.7–15.4)	13.4 (11.9–16)	**<0.001**
APTT (s)	26.8 (24–28.8)	27.1 (11.4–14.2)	25 (23.5–27.8)	25.3 (23.3–27.8)	**<0.001**
TT (s)	18.1 (17.1–19.3)	18.5 (17.8–19.1)	18.4 (17.9–19.1)	18.5 (17.7–19.2)	0.697
PT (s)	11 (10.6–11.4)	10.9 (10.6–11.5)	10.8 (10.3–11.4)	10.9 (10.4–11.4)	0.196

*Breath parameters*					
Minimum SaO_2_ (%)	90 (87–93)	88 (83–90)	82 (78–86)	69 (60–76)	**<0.001**
AHI	2.5 (1.5–3.6)	9.7 (6.6–12.4)	21.7 (18.3–25.9)	58.7 (44.6–72.7)	**<0.001**
ODI	3.1 (2.1–4.5)	9.5 (6.3–12.7)	22.7 (17.7–28.0)	59.2 (43.9–74.6)	**<0.001**
MAI	15.1 (10.8–24.8)	17.6 (12–25.9)	21.4 (15.0–30.0)	34.7 (19.2–53.2)	**<0.001**
CT90	0.06 (0.00–4.02)	2.19 (0.15–14.64)	5.20 (2.13–13.95)	21.92 (9.00–39.70)	**<0.001**

Data are presented as median with interquartile range. BMI, body mass index; PLT, platelet; MPV, mean platelet volume; PDW, platelet distribution width; APTT, activated partial thromboplastin time; TT, thrombin time; PT, prothrombin time; AHI, apnoea-hypopnea index; ODI, oxygen desaturation index; MAI, microarousal index; CT90, percentage of sleep time with oxygen saturation below 90%. The bold values indicate a statistical significance (*p*<0.05).

**Table 2:** Correlations between haematological data and breath-related parameters.

	PLT		MPV		PDW		APTT		PT		TT	
	*ß*	*P*	*ß*	*P*	*ß*	*P*	*ß*	*P*	*ß*	*P*	*ß*	*P*
Minimum SaO_2_ (%)	0.013	0.713	−0.025	0.472	−0.098	**0.004**	0.088	**0.013**	0.106	**0.003**	−0.008	0.817
AHI	−0.006	0.853	0.003	0.939	0.136	**<0.001**	−0.128	**<0.001**	−0.05	0.154	−0.038	0.281
ODI	−0.028	0.409	0.022	0.525	0.155	**<0.001**	−0.123	**0.001**	−0.045	0.212	−0.026	0.468
MAI	−0.002	0.962	−0.031	0.370	0.091	**0.008**	−0.017	0.634	−0.02	0.568	0.065	0.066
CT90	−0.067	0.056	−0.008	0.827	−0.092	**0.009**	−0.021	0.573	−0.048	0.189	0.022	0.549

PLT, platelet; MPV, mean platelet volume; PDW, platelet distribution width; APTT, activated partial thromboplastin time; TT, thrombin time; PT, prothrombin time; AHI, apnoea-hypopnea index; ODI, oxygen desaturation index; MAI, microarousal index; CT90, percentage of sleep time with oxygen saturation below 90%. The bold values indicate a statistical significance (*p*<0.05).

**Table 3:** Associations of OSA-related traits with the risk of coagulation.

	PDW			APTT			TT		
	OR	95% CI	*P*	OR	95% CI	*P*	OR	95% CI	*P*
*Model 1*									
Minimum SaO_2_ (%)	0.988	0.977–1.000	0.056	0.993	0.967–1.020	0.593	1.056	1.013–1.100	**0.01**
AHI	1.008	1.002–1.015	**0.015**	1.002	0.988–1.017	0.755	0.984	0.967–1.001	0.07
ODI	1.010	1.003–1.016	**0.003**	1.005	0.991–1.019	0.519	0.985	0.968–1.001	0.073
MAI	1.007	0.999–1.015	0.084	1.009	0.993–1.025	0.275	0.978	0.956–1.002	0.071
CT90	1.004	0.996–1.012	0.301	0.991	0.971–1.011	0.391	0.976	0.949–1.004	0.089

*Model 2*									
Minimum SaO_2_ (%)	0.990	0.978–1.002	0.093	0.994	0.967–1.021	0.645	1.053	1.010–1.098	**0.014**
AHI	1.007	1.001–1.014	**0.027**	1.002	0.987–1.017	0.799	0.985	0.968–1.003	0.098
ODI	1.009	1.003–1.016	**0.006**	1.004	0.990–1.019	0.565	0.986	0.969–1.003	0.106
MAI	1.006	0.999–1.014	0.107	1.009	0.993–1.025	0.299	0.979	0.956–1.003	0.085
CT90	1.004	0.996–1.012	0.347	0.991	0.970–1.011	0.364	0.977	0.950–1.005	0.107

*Model 3*									
Minimum SaO_2_ (%)	0.991	0.979–1.003	0.147	0.996	0.970–1.023	0.786	1.055	1.013–1.099	**0.011**
AHI	1.007	1.000–1.013	0.053	1.000	0.985–1.014	0.984	0.983	0.966–1.000	0.053
ODI	1.009	1.002–1.015	**0.008**	1.002	0.988–1.016	0.784	0.983	0.966–1.000	**0.05**
MAI	1.007	0.999–1.015	0.082	1.007	0.991–1.023	0.413	0.978	0.955–1.002	0.068
CT90	1.003	0.995–1.011	0.490	0.989	0.968–1.009	0.283	0.973	0.946–1.001	0.062

*Model 4*									
Minimum SaO_2_ (%)	0.989	0.977–1.001	0.074	0.994	0.968–1.021	0.662	1.051	1.008–1.095	**0.019**
AHI	1.008	1.001–1.015	**0.022**	1.002	0.987–1.017	0.797	0.986	0.969–1.004	0.117
ODI	1.009	1.003–1.016	**0.005**	1.004	0.990–1.018	0.587	0.987	0.970–1.004	0.123
MAI	1.007	0.999–1.014	0.103	1.010	0.994–1.026	0.237	0.978	0.955–1.002	0.073
CT90	0.977	0.950–1.005	0.104	0.991	0.971–1.011	0.381	0.977	0.950–1.005	0.104

*Model 5*									
Minimum SaO_2_ (%)	0.990	0.978–1.003	0.123	0.993	0.967–1.021	0.637	1.050	1.007–1.094	**0.021**
AHI	1.007	1.000–1.014	0.058	1.002	0.987–1.017	0.832	0.985	0.968–1.003	0.103
ODI	1.009	1.002–1.016	**0.009**	1.004	0.990–1.019	0.578	0.986	0.968–1.003	0.103
MAI	1.007	0.999–1.015	0.100	1.008	0.992–1.025	0.323	0.979	0.956–1.002	0.076
CT90	1.003	0.994–1.011	0.517	0.990	0.970–1.011	0.346	0.975	0.948–1.003	0.083

Model 1 was adjusted for age, BMI; model 2 was adjusted for variables included in model 1 and hypertension; model 3 was adjusted for age, waist-hip ratio, and hypertension; model 4 was further adjusted for smoking and drinking based on model 2; model 5 was adjusted for variables in model 4 and additional waist-hip ratio. PDW, platelet distribution width; APTT, activated partial thromboplastin time; TT, thrombin time; AHI, apnoea-hypopnea index; ODI, oxygen desaturation index; MAI, microarousal index; CT90, percentage of sleep time with oxygen saturation below 90%. The bold values indicate a statistical significance (*p*<0.05).

## Data Availability

The original clinical data used to support the findings of this study are available from the corresponding author upon request.

## Additional Points

(i) PDW increased, and APTT decreased with the increase in OSA severity. (ii) PDW and APTT correlate with OSA-related traits. (iii) OSA increased the risk of an abnormal PDW. (iv) Nonlinear relationships of OSA-related traits with PDW and APTT were discovered. (v) The abnormal alteration of platelets and the coagulation system in OSA might contribute to the cardiovascular complications.
